# A Research Communication Brief: Gluten Analysis in Beef Samples Collected Using a Rigorous, Nationally Representative Sampling Protocol Confirms That Grain-Finished Beef Is Naturally Gluten-Free

**DOI:** 10.3390/nu9090936

**Published:** 2017-08-25

**Authors:** Shalene H. McNeill, Amy M. Cifelli, Janet M. Roseland, Keith E. Belk, Dale R. Woerner, Kerri B. Gehring, Jeffrey W. Savell, J. Chance Brooks, Leslie D. Thompson

**Affiliations:** 1 National Cattlemen’s Beef Association, Centennial, CO 80112, USA; smcneill@beef.org; 2 Nutrient Data Laboratory, United States Department of Agriculture/Agricultural Research Service, Beltsville, MD 20705, USA; janet.roseland@ars.usda.gov; 3 Department of Animal Sciences, Colorado State University, Fort Collins, CO 80523, USA; keith.belk@colostate.edu (K.E.B.); dale.woerner@colostate.edu (D.R.W.); 4 Department of Animal Science, Texas A&M University, College Station, TX 77843, USA; kbgehring@tamu.edu (K.B.G.); j-savell@tamu.edu (J.W.S.); 5 Department of Animal and Food Sciences, Texas Tech University, Lubbock, TX 79415, USA; chance.brooks@ttu.edu (J.C.B.); leslie.thompson@ttu.edu (L.D.T.)

**Keywords:** beef, gluten, celiac disease, gluten sensitivity, nutrient data

## Abstract

Knowing whether or not a food contains gluten is vital for the growing number of individuals with celiac disease and non-celiac gluten sensitivity. Questions have recently been raised about whether beef from conventionally-raised, grain-finished cattle may contain gluten. To date, basic principles of ruminant digestion have been cited in support of the prevailing expert opinion that beef is inherently gluten-free. For this study, gluten analysis was conducted in beef samples collected using a rigorous nationally representative sampling protocol to determine whether gluten was present. The findings of our research uphold the understanding of the principles of gluten digestion in beef cattle and corroborate recommendations that recognize beef as a naturally gluten-free food.

## 1. Introduction

Experts recognize fresh meat such as beef as a naturally gluten-free food that is recommended as part of a healthful gluten-free diet [1,2,3,4] Beef is an important source of 10 essential nutrients including protein and key micronutrients such as iron, zinc, and B-vitamins, which are nutrients of concern for those following a gluten-free diet [5,6,7,8,9,10]. Questions have recently been raised about whether beef from conventionally-raised, grain-finished cattle may contain gluten. To date, basic principles of ruminant digestion have been cited in support of the prevailing expert opinion that beef is inherently gluten-free [11]. Although wheat, barley, and rye are common gluten-containing feed ingredients in conventional, grain-finished cattle feeds, it is well accepted that gluten proteins are hydrolyzed into individual amino acids during the ruminant digestive process. While there is general scientific consensus based on well-accepted animal physiology that meat from grain-finished beef cattle does not contain gluten, this has not been scientifically validated using current analytical methods for evaluating the gluten content of foods. Thus, gluten analysis was conducted in beef samples collected using a rigorous nationally representative sampling protocol. The findings confirm the understanding of the principles of gluten digestion in beef cattle and corroborate recommendations that recognize beef as a naturally gluten-free food.

Celiac disease affects an estimated 1% of the population in the United States, while non-celiac gluten sensitivity is estimated to affect another 0.6% to 6% [12,13]. Thus, up to 7% of people in the U.S. may benefit from a gluten-free diet. This gluten analysis in beef provides confidence for the large number of individuals following a gluten-free diet.

## 2. Materials and Methods 

### 2.1. Sampling Protocol

In order to provide accurate nutrition information to health professionals, the food industry, and consumers, the national Beef Checkoff Program and the USDA Agricultural Research Service have collaborated to conduct research resulting in updated nutrient composition data for beef retail cuts published in the USDA National Nutrient Database for Standard Reference [14]. To accurately obtain research samples representing beef retail cuts in the U.S., a rigorous nationally representative sampling protocol was developed by nutrition and meat scientists at three universities in collaboration with USDA Nutrient Data Laboratory statisticians [15,16,17,18].

These experts identified a nationally representative sample of 164 beef carcasses at seven meat packing plants in six different regions. A statistically appropriate sample was selected to represent the proper proportions of yield grade, quality grade, breed, genetic type and geographic location. The carcasses were sent to the three collaborating universities for fabrication into retail cuts. Raw and cooked samples were homogenized, and composites were made for each retail cut. A chart illustrating the study sample protocol is published elsewhere [19]. 

### 2.2. Nutrient Analysis

Comprehensive nutrient analysis was conducted on the raw and cooked composite samples for the retail beef cuts. This included analysis for total protein and amino acids, total fat and fatty acids, cholesterol, minerals such as iron, selenium, and zinc, as well as vitamins including retinol, B-vitamins, choline, vitamin D, and vitamin E. Validated nutrient analysis methods and quality control techniques were performed throughout the study to ensure accurate nutrient data, as has been previously described [13,14,15,16]. The results of this comprehensive nutrient analysis served to update the USDA’s National Nutrient Database for Standard Reference [20].

## 3. Results

In March 2015, gluten analysis was conducted on archived samples retained from the beef research described above. A total of 17 composite samples representing 17 retail beef cuts were sent to an independent laboratory for gluten analysis. Food Safety Net Services performed the gluten analysis using Veratox^®^ (Neogen Food Safety, Lansing, MI, USA) for Gliadin R5, a validated sandwich enzyme-linked immunoassay (S-ELISA) test (Neogen Corporation and the University of Nebraska Food Allergy Research and Resource Program, Lincoln, NE, USA, 2012) distributed by Neogen^®^ (Neogen Food Safety, Lansing, MI, USA) Corporation. 

The gluten analysis results for each of the 17 composite beef samples were below the limit of detection for this test that is equivalent to less than 5 ppm of gluten (see Table 1). According to the FDA’s 2013 Gluten-Free Labelling regulations, a food that is inherently free of gluten may be labelled as “gluten-free” [21,22,23].

## 4. Discussion

These findings confirm that today’s fresh beef supply from conventionally-raised cattle—the predominant type sold in grocery stores—does not contain measurable levels of gluten, and can be included in a gluten-free diet. This evidence may help individuals with gluten-related conditions avoid unnecessary dietary restriction and can provide important nutritional benefits due to the micronutrients found in beef such as iron, zinc, and B-vitamins.

A gluten-free diet is currently the only safe treatment for individuals diagnosed with celiac disease [24,25]. Left untreated, this genetic autoimmune disorder is associated with a wide range of symptoms, nutrient deficiencies, and serious complications. In susceptible individuals, the ingestion of the gluten protein in wheat, barley, rye, and crossbreeds of these grains damages the villi in the small intestine. This damage to the small intestine typically results in the malabsorption of vital nutrients such as iron, zinc, and B-vitamins. Anemia resulting from the malabsorption of iron and vitamin B_12_ is of particular concern for those with celiac disease. Avoiding or correcting nutrient deficiencies that are often present with celiac disease is a key focus of medical nutrition therapy. 

It is well documented that those following a gluten-free diet are at increased risk of multiple vitamin and mineral deficiencies for a number of reasons [7,8,9]. For example, gluten-free flours and grain products such as breads, pastas, and cereals are not subject to the same enrichment and fortification standards as wheat-based products. Thus, many gluten-free grain products contain lower levels of iron and B-vitamins such as thiamin, riboflavin, niacin, and folate. This further increases the risk of iron and B-vitamin deficiencies in individuals following a gluten-free diet.

## 5. Conclusions

Knowing whether or not a food contains gluten is vital information for individuals with celiac disease and non-celiac gluten sensitivity. The approach described in this report can serve as a model for others interested in substantiating the gluten-free nature of their products. The publication of results from gluten analyses such as this can help to further inform health professionals and the food industry and ultimately benefit those who must avoid gluten in their diets.

To our knowledge, this is the first effort to conduct gluten analysis in a nationally representative sample of beef. The rigorous sampling protocol and validated enzyme-linked immunoassay used for this analysis provides scientific evidence to support current recommendations that recognize beef as an inherently gluten-free food that can be enjoyed in a healthful gluten-free diet. This understanding is important since beef is a source of many vital nutrients such as iron, zinc, and B-vitamins that are of concern for those following a gluten-free diet. Encouraging gluten-restricting individuals to enjoy beef as part of a healthful gluten-free diet may reduce unnecessary dietary restriction, and improve diet satisfaction and nutrient adequacy. 

## Figures and Tables

**Table 1:** Gluten content of composite samples from 17 retail beef cuts.

Retail Beef Cuts (Raw Composite Samples)	Gluten Analysis Results (ppm *) (Lowest Limit of Detection = 5 ppm)
Brisket, Flat Half	<5 ppm
Clod Roast	<5 ppm
Stew	<5 ppm
Denver Cut	<5 ppm
Underblade Roast	<5 ppm
Country Style Ribs	<5 ppm
America’s Beef Roast	<5 ppm
Chuck Eye Steak	<5 ppm
Top Blade Steak	<5 ppm
Mock Tender Steak	<5 ppm
Short Ribs	<5 ppm
Ribeye Steak (bone-in)	<5 ppm
Ribeye Steak (boneless)	<5 ppm
Inside Skirt Steak	<5 ppm
Outside Skirt Steak	<5 ppm
Porterhouse Steak	<5 ppm
T-Bone steak	<5 ppm

* ppm = parts per million.
